# Integration of amyloid-β oligomerization tendency as a plasma biomarker in Alzheimer's disease diagnosis

**DOI:** 10.3389/fneur.2022.1028448

**Published:** 2023-01-17

**Authors:** Jung-Min Pyun, Young Chul Youn, Young Ho Park, SangYun Kim

**Affiliations:** ^1^Department of Neurology, Soonchunhyang University Seoul Hospital, Soonchunhyang University College of Medicine, Seoul, Republic of Korea; ^2^Department of Neurology, Chung-Ang University College of Medicine, Seoul, Republic of Korea; ^3^Department of Neurology, Seoul National University College of Medicine and Seoul National University Bundang Hospital, Seongnam-si, Gyeonggi-do, Republic of Korea

**Keywords:** Alzheimer's disease, blood-based biomarker, amyloid-β, oligomerization tendency, diagnosis, neuropsychological test, brain MRI

## Abstract

**Introduction:**

There has been significant development in blood-based biomarkers targeting amyloidopathy of Alzheimer's disease (AD). However, the guidelines for integrating such biomarkers into AD diagnosis are still inadequate. Multimer Detection System-Oligomeric Amyloid-β (MDS-OAβ) as a plasma biomarker detecting oligomerization tendency is available in the clinical practice.

**Main text:**

We suggest how to interpret the results of plasma biomarker for amyloidopathy using MDS-OAβ with neuropsychological test, brain magnetic resonance imaging (MRI), and amyloid PET for AD diagnosis. Combination of each test result differentiates various stages of AD, other neurodegenerative diseases, or cognitive impairment due to the causes other than neurodegeneration.

**Discussion:**

A systematic interpretation strategy could support accurate diagnosis and staging of AD. Moreover, comprehensive use of biomarkers that target amyloidopathy such as amyloid PET on brain amyloid plaque and MDS-OAβ on amyloid-β oligomerization tendency can complement to gain advanced insights on amyloid-β dynamics in AD.

## Introduction

The advances in the development of blood-based biomarkers targeting amyloidopathy of Alzheimer's disease (AD) have been remarkable in showing high performances of predicting clinical AD and central AD pathology (1–3). Although current blood-based biomarkers have the issues to improve, their clinical application is promising based on the strength of low cost, non-invasiveness, and the ease of performance (4). Nevertheless, the guidelines for interpretation of blood-based biomarker with other AD diagnostic tools are scarce, and they would be meaningful in terms of fulfilling the broad interest from primary physicians to AD specialists and from the clinical practice to research field facing patients with progressive cognitive impairment.

Multimer Detection System-Oligomeric Amyloid-β (MDS-OAβ), one of the blood-based biomarkers detecting amyloidopathy, measures the oligomerization tendency of amyloid-β (Aβ) in blood, and its high predicting performances for central amyloidopathy and clinical AD have been reported (5–8). MDS-OAβ was approved by the Ministry of Food and Drug Safety (MFDS) and National Evidence-based healthcare Collaborating Agency (NECA) of Korea and is being used in the clinical practice. At this point, we would like to suggest how to integrate its results with other diagnostic tools including neuropsychological test, brain magnetic resonance imaging (MRI), and amyloid positron emission tomography (PET) for AD diagnosis.

## Main text

MDS-OAβ, a chemiluminescence immunoassay, detects oligomerization tendency of plasma using epitope-overlapping antibodies specific for N-terminus of Aβ (5, 6). Plasma is spiked with synthetic Aβ and incubated. This pretreated plasma is loaded into the 96-well microplate coated with the capture antibodies during which heterogenous forms of Aβ are captured. After washing, detection antibodies are added and Aβ oligomers, known as the most neurotoxic form, are selectively detected over Aβ monomers. MDS-OAβ could differentiate between AD dementia (ADD) and cognitively normal group with a sensitivity of 100% and specificity of 92% in the tester-blinded study with a MFDS-approved protocol (7).

### Interpretation of MDS-OAβ with neuropsychological test and brain MRI

Previous study showed that MDS-OAβ was higher in subjects with mild cognitive impairment due to AD (AD-MCI) and ADD compared to cognitively normal subjects. MDS-OAβ was lower in advanced stages such as stages in clinical dementia rating of 2–3 than mild dementia or MCI states (6–8). This finding accords with the hypothesis that amyloidopathy progresses in the early stage of AD and reaches plateau state in the advanced stage (9). Namely, whereas the progression rate of amyloidopathy stays stable, the oligomerization tendency of Aβ detected by MDS-OAβ could decrease in the advanced stage. Because MDS-OAβ measures dynamic changes after spiking of synthetic Aβ in plasma, MDS-OAβ reflects plasma milieu of patients and indicates the upstream biomarker of amyloidopathy. Many upstream biomarkers show the dynamic changes of increase in the early stage of disease and decrease in the advanced stages of disease (10). Additionally, high MDS-OAβ was associated with atrophy bilateral temporal, amygdala, parahippocampal, lower parietal lobe, left cingulate, and precuneus area on brain MRI, which corresponds to AD pattern (8).

Patients with positive MDS-OAβ, cognitive impairment with insidious onset and slow progression, and AD-compatible atrophy on brain MRI are most likely to have AD. Rarely, other neurodegenerative diseases with concomitant amyloidopathy could be considered. Dementia with Lewy bodies (DLBs) and Parkinson disease dementia (PDD) have main pathology of α-synucleinopathy and could accompany with amyloidopathy with various extent (11, 12). In frontotemporal dementia (FTD), amyloidopathy could be present with main pathologic protein such as tau or TAR DNA-binding protein 43 (TDP-43) (12, 13). Also, vascular dementia (VD) and normal pressure hydrocephalus (NPH) could show amyloidopathy (12, 14). Limbic-predominant age-related TDP-43 encephalopathy (LATE) could have amyloidopathy along with neurodegeneration by TDP-43 (15).

Patients with positive MDS-OAβ, cognitive impairment, and normal brain MRI could be in the early stage of AD during which structural changes on brain MRI are not clear. Rarely, other causes including depression, vitamin B12/folate deficiency, electrolyte imbalance, and poor general medical condition could cause cognitive impairment and amyloidopathy to co-exist.

Patients with positive MDS-OAβ, normal cognition, and AD-compatible atrophy on MRI could have preclinical AD with progressive amyloidopathy without clinical manifestation. Rarely, other neurodegenerative diseases such as DLB, FTD, NPH, VD, PDD, or LATE mixed with amyloidopathy could be considered.

Patients with positive MDS-OAβ, normal cognition, and normal brain MRI could have preclinical amyloidopathy or they are at a high risk of AD. In case of negative MDS-OAβ, cognitive impairment, and AD-compatible atrophy on MRI, other neurodegenerative diseases such as DLB, FTD, NPH, VD, PDD, or LATE could be considered.

Patients with negative MDS-OAβ, cognitive impairment, and normal brain MRI could attribute the cognitive impairment to other causes including depression, vitamin B12/folate deficiency, electrolyte imbalance, or poor general medical condition.

Patients with negative MDS-OAβ, normal cognition, and AD-compatible atrophy on MRI could have other preclinical neurodegenerative diseases such as DLB, FTD, NPH, VD, PDD, or LATE.

Patients with negative MDS-OAβ, normal cognition, and normal brain MRI could be the conditions without AD-suspicious pathological evidence (Table 1).

**Table 1:** Interpretations of MDS-OAβ, neuropsychological test, and brain MRI.

**MDS-OAβ**	**Neuro-** **psychological test**	**Brain MRI**	**The most probable diagnosis**	**Remarks**	**Recommend**
Positive	Cognitive impairment	AD-compatible atrophy	AD	Rarely, other NDDs (DLB, FTD, NPH, PDD, VD, LATE, etc.) + amyloidopathy	Treatment of AD/NDDs & MDS-OAβ follow-up
		Normal	Early stage of AD	Rarely, cognitive impairment due to other causes (depression, vitamin B12/folate deficiency, electrolyte imbalance, poor general medical condition, etc.,) + amyloidopathy	Treatment of AD/other causes & AD risk factor control & MDS-OAβ follow-up
	Normal	AD-compatible atrophy	Preclinical AD	Other preclinical NDDs (DLB, FTD, NPH, PDD, VD, LATE, etc.) + amyloidopathy	AD/NDDs risk factor control & MDS-OAβ/NPT follow-up
		Normal	Preclinical amyloidopathy		AD risk factor control & MDS-OAβ/NPT follow-up
Negative	Cognitive impairment	AD-compatible atrophy	Other NDDs (DLB, FTD, NPH, PDD, VD, LATE, etc.)		Treatment of NDDs & MDS-OAβ follow-up
		Normal	Cognitive impairment due to other causes (depression, vitamin B12/folate deficiency, electrolyte imbalance, poor general medical condition, etc.,)		Treatment of other causes
	Normal	Abnormal	Other preclinical NDDs (DLB, FTD, NPH, PDD, VD, LATE, etc.,)		NDDs risk factor control & NPT follow-up
		Normal	Normal		With concern about cognitive impairment, MDS-OAβ/NPT follow-up

### Interpretation of MDS-OAβ with amyloid PET

Previous study reported that MDS-OAβ could predict amyloid PET positivity with area under the receiver operating characteristic curve value of 0.855 (16). MDS-OAβ and amyloid PET detect amyloidopathy with different biomarkers. MDS-OAβ measures oligomerization tendency instead of measuring concentration of each Aβ species or related peptides, but amyloid PET ligands react to insoluble amyloid fibril incorporated in plaque. Since both biomarkers have different characteristics and dynamics, their interpretation requires caution.

Patients with positive MDS-OAβ and positive amyloid PET could be most likely to have AD.

Patients with positive MDS-OAβ and negative amyloid PET could present amyloidopathy in progress without manifestation of amyloid plaques. These unmatched cases require further observation and studies regarding their different characteristics of patients and biomarkers itself. Issues to think are that ligands of amyloid PET are reactive to fibrillary form Aβ and low binding affinity to diffuse plaques. Moreover, although visual assessment of amyloid PET dichotomizes the patients into positive or negative, from the perspective of continuous spectrum, borderline negative cases near the cut-off values and definite negative cases far from the cut-off values should be differentiated based on the degree of amyloidopathy.

Patients with negative MDS-OAβ and positive amyloid PET could indicate advanced AD in case of cognitive impairment, where amyloidopathy is in plateau state (17); otherwise, preclinical AD is also possible in case of no cognitive impairment (18). Researches showed that the prevalence rate of cognitively normal elderly with positive amyloid PET reaches 10–30%, and each individual has different starting points of amyloid deposition (19, 20). Therefore, positive amyloid PET does not always indicate advanced stage or longer duration of disease (17). Patients with negative MDS-OAβ and negative amyloid PET are not likely to have amyloidopathy. In case of cognitive impairment, causes other than AD should be considered.

Empirically, some chemotherapeutic agents, immunotherapeutic agents, or passive immunization could lower the value of MDS-OAβ.

## Discussion

Even though the advanced diagnostic tools may present high performance, practical use in the clinical field requires proper interpretation to be of real value. This systematic interpretation suggests that MDS-OAβ could support more accurate diagnosis and staging of AD when combined with other biomarkers and provide helpful clues in diverse matches of clinical manifestation and test results, even in atypical presentation due to mixed pathologies. MDS-OAβ as a unique technique detecting oligomerization tendency measures the key neurotoxic process of AD, and therefore, MDS-OAβ would be suitable for diagnosis and disease monitoring with multiple re-tests possible due to low cost and invasiveness. Additionally, understanding factors affecting values of MDS-OAβ could provide clues about plasma milieu of patients with AD and might be useful in disease intervention in the future.

**Figure 1:**
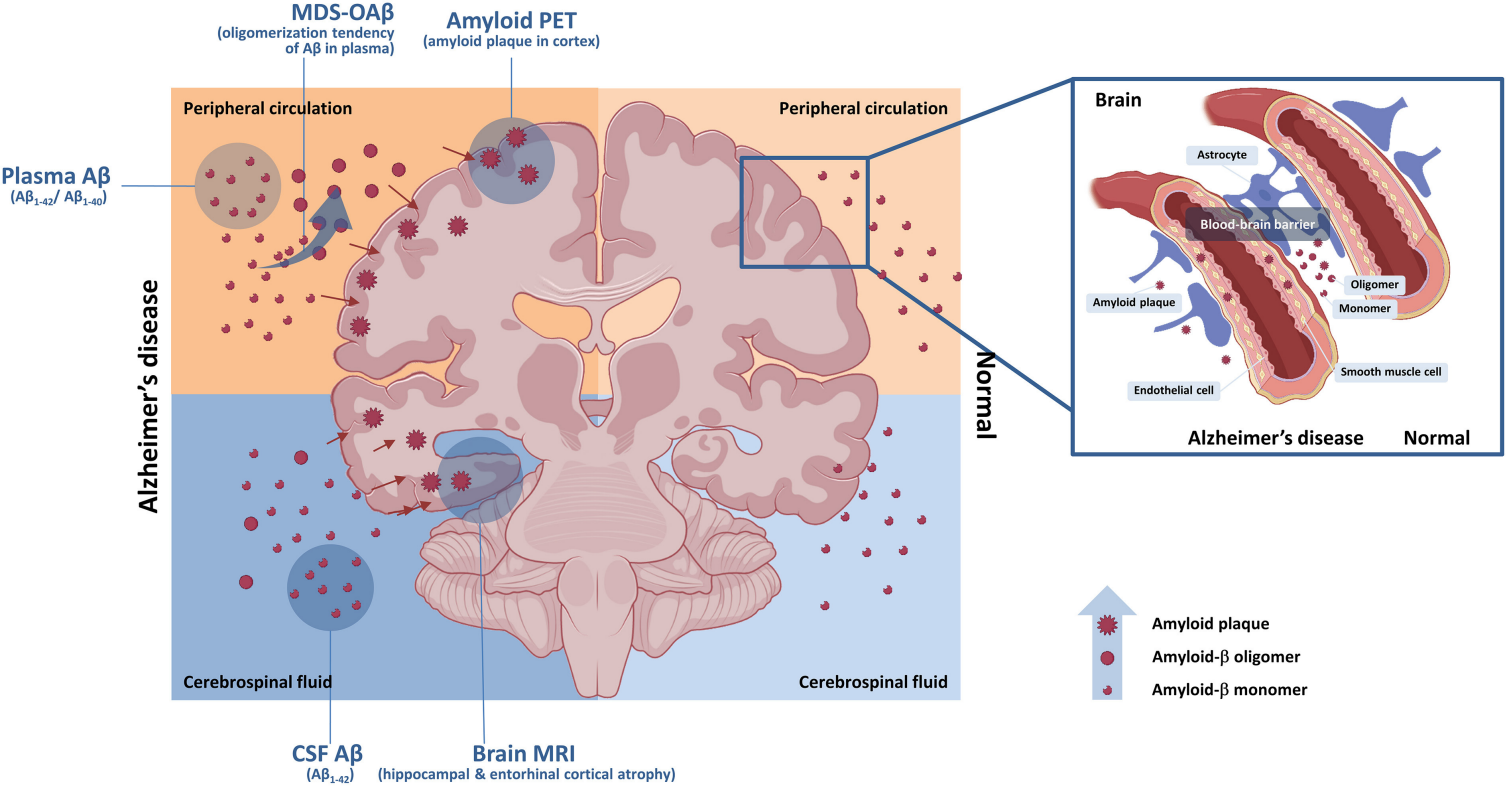
Central and peripheral amyloid-β and biomarkers. Central and peripheral Aβ can be detected by various biomarkers. Aβ oligomerization tendency in plasma can be detected by MDS-OAβ. Concentration of Aβ in CSF can be measured by CSF study with spinal tapping. Accumulation of Aβ plaque in brain can be evaluated by amyloid PET. Impaired blood–brain barrier can contribute to acceleration of amyloid pathology. Aβ, amyloid-β; CSF, cerebrospinal fluid; MDS-OAβ, Multimer Detection System-Oligomeric Amyloid-β; MRI, brain magnetic resonance imaging; PET, positron emission tomography.

Collective interpretation of plasma biomarkers of AD should be avoided because each plasma biomarker of AD has different mechanisms and targets. Additionally, further autopsy studies and longitudinal data can strengthen evidence base for the interpretation. However, strategies of their clinical integration shall be essential in the era of immediate application of plasma biomarker of AD. Moreover, comprehensive use of various biomarker tools related to amyloidopathy such as amyloid plaque using PET, oligomerization tendency using MDS-OAβ, plasma, and CSF Aβ concentration could complement to gain advanced insights on Aβ dynamics in AD (Figure 1).

## Data availability statement

The original contributions presented in the study are included in the article/supplementary material, further inquiries can be directed to the corresponding author.

## Author contributions

J-MP was a major contributor in writing the manuscript. YY and YP revised the manuscript. SK designed the work and revised the manuscript. All authors read and approved the final manuscript.
